# “You Can’t Look at an Orange and Draw a Banana”: Using Research Evidence to Develop Relevant Health Policy in Ghana

**DOI:** 10.9745/GHSP-D-21-00693

**Published:** 2022-09-15

**Authors:** Ayaga A. Bawah, Adriana A.E. Biney, Pearl Kyei

**Affiliations:** a University of Ghana, Accra, Ghana.

## Abstract

We examined factors that either enabled or inhibited the process of evidence-based decision making regarding health policy in Ghana. We conducted qualitative interviews with 2 major groups of stakeholders: health policy and systems research producers (research producers [RPs]) and policy makers (PMs). In-depth interviews were conducted with 12 RPs, who were representatives from 11 health policy and systems research institutions; and 12 PMs working in various national health-related agencies, ministries, and departments. We analyzed the data using the thematic analysis approach. Interview results showed 5 recurring themes in their discussion of enablers and inhibitors of the evidence-to-policy process: (1) the quality, relevance, and quantity of available research evidence; (2) how findings are communicated to PMs; (3) the strength of relationships between RPs and PMs; (4) available structures that promote evidence-based policy making; and (5) the political context in which research and policy making occurs. These findings point to some specific areas for further collaboration and communication among Ghanaian stakeholders to ensure that appropriate health policies are developed from an evidence base.

## Abstract (precis)

We explored inhibitors and enablers of using health policy and systems research to inform the policy process in Ghana. The findings suggest a myriad of factors influencing evidence-based policy development, including the strength of the relationships between policy makers and research producers.

## Teaser

**Key Findings:**

- Research producers (RPs) and policy makers (PMs) identified similar enablers and inhibitors of evidence-based policy making including having high-quality evidence that is relevant to current policies under consideration.
- Building relationships between RPs and PMs at the beginning of the research process will help build trust, promote mutual understanding, ensure that research is relevant to policy priorities, encourage evidence uptake, and facilitate the research-to-policy process.

**Key Implications:**

- RPs and PMs require platforms to enable collaboration and communication on research studies to facilitate program implementation and evidence-based policy making.
- RPs need additional support to engage in policy dialogue platforms and in working in political contexts to communicate their research in ways that are readily understood.

## INTRODUCTION

Using robust research evidence to inform public health policy ensures the most equitable population health gains.^1^ However, little attention has been given to the mechanics underlying policy makers’ (PMs’) use of research results to inform policy.^2^ As a National Research Council report states, “when science has something to offer, it should be on the policy table.”^3^

While there may seem to be a natural trajectory from research to policy, this process is rarely straightforward. The decision-making process of using research to inform policy tends to be iterative rather than unilinear in nature. For research producers (RPs) and PMs to find some synergy and thus facilitate the transition from research to policy, researchers should understand the mechanics of the decision-making process, just as PMs need to have an understanding of the conceptual basis of the research process.^4^ La Brooy and Kelaher^4^ note that getting research evidence into practice is not like shelving products in a retail shop and expecting prospective buyers to pluck them off the shelves—there must be interactive engagement between RPs and PMs. However, low- and middle-income countries (LMICs) face particular challenges with this process, including limited access to evidence, the exclusion of PMs and implementers from setting the research agenda, weak health care systems, and limited PM capacity to interpret research findings.^5^^–^^7^ Further, limited interactions among RPs, PMs, implementers, and other stakeholders tend to constrain the use of research in policy updates.^5^^,^^8^^–^^10^

Ghana poses somewhat of an exception to this typical scenario, with its long tradition of using research evidence to inform policy.^11^^,^^12^ For example, the country adopted the vitamin A policy based on evidence from a demographic surveillance site that showed health improvements among children aged 5 years and younger who were given vitamin A supplements.^11^ Under Ghana’s National Malaria Control Program, research and modeling of scenarios led to policies that have resulted in reductions in deaths due to malaria of children under 5.^13^ Additionally, the Community-Based Health Planning and Services initiative and the National Health Insurance Scheme represent health policies originating in feasibility studies carried out at national health research institutes.^14^^–^^16^ Both national programs were based on empirical health policy research that identified effective interventions, which, over time, were adopted into policies and are now being implemented at scale to improve health. Ghana has used the process of testing and refining service delivery strategies, engaging with communities, and maintaining dialogue between RPs and PMs at all stages of the research-to-policy process—from the conception of an idea throughout the phases of research until adoption as policy.^13^^–^^18^

Despite Ghana’s successes, the picture is not so rosy. The country does not have a clear research-to-policy structure in place, nor has it assessed the facilitators of and barriers to the process. As Rycroft-Malone affirms, “often practice lags behind what is known to be current best practice”^19^ for various reasons. For instance, not enough institutions have the capacity and wherewithal to carry out wide-ranging research and achieve the successes of these national health institutes. Large-scale project funding is not always available to support implementing agencies in replicating such research. These and other barriers, as well as factors that facilitate the research-to-policy process, need to be explored.

Ghana does not have a clear research-to-policy structure in place, nor has it assessed the enablers and inhibitors of the process.

Drawing from the Promoting Action on Research Implementation in Health Services (PARIHS) framework,^19^^–^^22^ we present an evaluation of Ghana’s experience generating and using health policy and systems research (HPSR) results to inform policy decisions. From the perspectives of a range of policy officials, we assess factors affecting the generation of evidence^1^^,^^2^ and the uptake of research to inform health policy decisions in the country. We also examine the demand for and culture of using evidence to make decisions among Ghanaian PMs. Finally, we aim to equip both health research and policy institutions with the knowledge to mitigate challenges with the process and ways to enhance prospects for research utilization in the future.

## METHODS

### Study Procedure, Setting, and Participants

We conducted this research as part of a multicountry study of the research-to-policy process in Ghana, Ethiopia, and Mozambique, commissioned by the World Health Organization (WHO) Alliance for Health Policy and Systems Research (AHPSR).^23^ We used a qualitative research design to explore the aforementioned study aims and conducted in-depth and semistructured interviews with RPs and PMs.

The team identified potential study participants based on their knowledge of relevant institutions and individuals working on health policy in the country. We distributed official letters to their targeted participants’ offices and followed up with a series of correspondence (through email, phone calls, or in-person visits) to set up the interviews.

We selected the RPs from public and private research institutions (university institutes, research institutes under the Ministry of Health/Ghana Health Service, and a national research organization) possessing 2 main characteristics: (1) it is a domestic/Ghana-based institution; and (2) it had conducted HPSR in the 3 years preceding the study. The second requirement served to ensure the institution was established in the field as a health research-producing unit. Our targeted study participants were heads of the institutions, who either granted the interview or selected a representative to be interviewed. A total of 12 RPs drawn from 11 of 15 identified HPSR institutions in Ghana were interviewed with a semistructured qualitative guide. There were 8 men and 4 women with an average of 19 years of HPSR experience.

The research team generated an initial list of 26 PMs based on participants’ positions at relevant governmental and international organizations and agencies involved in Ghana’s health policy. Of the 26 proposed PMs, 12 (10 men and 2 women) were interviewed. The interviewed PMs included officials with health policy and systems research expertise from the Ministry of Health, Ghana Health Service, Ghana Statistical Service, National Development Planning Commission, and United Nations Population Fund, as well as a member of Parliament. Semistructured interview guides were also administered to the 12 PMs during their in-depth interviews. The remaining RPs and PMs could not be interviewed because either they were unavailable or their agencies had bureaucratic requirements that were not feasible within the study period.

We used in-depth interview guides for RPs and PMs initially developed by AHPSR researchers and slightly revised by the Ghana research team to fit the Ghanaian context (Supplement). The tools were based on the WHO AHPSR study objectives and considered the cyclical relationship between RPs and PMs.^23^ The RPs’ guides contained questions about their institutions’ influence on the policy process and its enablers, structures at their institutions that enable policy-relevant research, and their institutions’ contributions to national/international health policies. PMs’ guides included questions about their use of research evidence to inform policy decisions, the culture of evidence-based health policy in Ghana and how it has changed over time, and their thoughts on enablers of and barriers to using evidence to inform policy.

The Ghana research team consisted of the principal investigator providing oversight for the entire project; 2 researchers supervising data collection and carrying out analysis; and 5 interviewers (1 with a doctoral degree and 4 doctoral candidates). During a 2-day training session, the principal investigator and 2 supervisory researchers briefed the interviewers on the overall study objectives and use of the interview guides. This rigorous session ensured they were familiar with the guide, understood the questions, and could obtain reliable information from study participants.

### Data Collection

The team conducted 24 in-depth qualitative interviews with RPs and PMs.

The interviews were conducted between February and June 2020 in person and, following the detection of COVID-19 cases in Ghana in March 2020, also virtually. All in-person interviews were conducted at the workplaces of the study participants. In-depth interviews with RPs and PMs averaged 55 minutes and 57 minutes, respectively.

### Data Analysis

All interviews were conducted in English, audio-recorded with consent from study participants, and transcribed exactly as stated during the interviews to reduce any bias in reporting participants’ statements. The interviewers also checked the final versions of the transcripts for accuracy. RP and PM transcripts were analyzed separately using Atlas.ti, Version 7. Analysis involved the application of deductive and inductive codes to inter-viewees’ responses to indicate either an enabler or an inhibitor of the use of research in policy making. We applied core components (quality of research evidence, nature of the context, and facilitation) of the original PARIHS framework as a broad guide for deductive coding.^19^^–^^22^ Inductive codes and themes were also linked to these 3 components. For example, the theme on modes of communicating findings was related to facilitation where the facilitator (in this case, the PM) encouraged RPs to develop skills to communicate their research.

A lead qualitative researcher initiated the analysis by developing initial codes and grouping these into subthemes (basic themes and organizing themes), which then fell under 1 of 5 global themes, based on her review of the transcripts. Additionally, 2 interviewers contributed to inter-coder reliability by also identifying codes and themes from the transcripts and checking them against the lead qualitative researcher’s identified codes. This process generated 3 new codes under the existing themes. We merged codes (deductive and/or inductive) into relevant basic, organizing, and global themes^24^^,^^25^ and identified and presented significant quotes from the transcripts. For example, the code “academia-implementer disconnect” was generated from the transcript of an RP in an academic research institute. This code was grouped under the organizing theme “relationship with PMs” linked to the third global theme “strength of relationships” and discussed as a barrier.

### Ethics Approval

Ethical clearance for the study was obtained from the Ethics Review Committee of the Ghana Health Service (ERC-GHS) with Protocol ID number: GHS-ERC 012/11/19. All participants gave their informed consent to participate in the interview and have the interview audio-recorded.

## RESULTS

The 5 overarching or global themes that emerged from the interviews align with the study’s main research objective: identifying enablers and in-hibitors of the integration of evidence into health policy decision making. The RPs and PMs were from institutions known for their expertise in health research and policy and thus provided relevant perspectives on the topic. In interviews with both groups, 3 broad themes of enabling or in-hibitorsfactors emerged: (1) quality, relevance, and quantity of research evidence; (2) how findings are communicated to PMs; and (3) strength of relationships between RPs and PMs. PMs raised 2 additional themes: (4) available structures that promote evidence-based policy; and (5) political context and conditions in which research and policy making occur. These 5 themes highlight the “dynamic and simultaneous relationship”^19^ between research, context, and the facilitation process leading to the successful implementation of evidence.

### Relevance, Quality, and Quantity of Research

The first key factor in the use of evidence for policy making is the evidence itself. Both RPs and PMs described how the relevance, quality, and quantity of available evidence contributed to its uptake.

The first key factor in the use of evidence for policy making is the evidence itself.

#### Quality and Credibility of Research and RP

RPs believed that the quality of the research, how compelling the evidence is, and the trustworthiness of the data were all important enablers in translating research results to policy.

*I think the key thing is the strength of the evidence and how relevant it is to the setting in which the study is conducted.* —RP-4

The research must offer sufficient evidence or information to appropriately inform PMs on the issues.

Other RPs indicated that the credibility of the research is intrinsically linked to the reputations of RPs or the organization that generated it.

*Where the information is coming from has also been [a] key part [of whether it is used], but also the quality of the research. So, if the PMs trust and believe that this is an institution that is reputed for doing a particular research … [it] all influences the ability to enable a decision to be made or influence the policy maker.* — RP-6

Further, 6 interviewees discussed the credibility of HPSR institutions as an important enabler of the research-to-policy process. Factors giving institutions credibility included the type and mandate of an institution, diversity and breadth of staff expertise and capacity, level and type of funding the institution attracts, the institution’s ability to advocate for causes, and the institution’s networks.

RPs noted that international partners assess the quality of a study design before awarding funds. Research commissioned by international donors was often deemed more rigorous and therefore deserving of attention than that funded domestically.

*When the research project is funded by international donors, [PMs] feel that already the rigor of the research has been looked at by the international partner … they believe that the quality is good.* —RP-2

PMs generally agreed that research quality was of interest to them. They saw the credibility and trustworthiness of RPs and research produced as especially important enablers of getting and using evidence for policy. For example, students’ research would not be valued as much as research produced by qualified academics.

*You know, trust is one big factor. If I get research information from, say, a first-degree student and then that of a professor, which one will I depend on? It’s [the] credibility. That’s very important. How credible is your source?* —PM-2

Inversely, a key inhibitor that PMs noted was the lack of credibility of the source of evidence. PMs also noted that the feasibility and actionability of any recommendations RPs provided were important to them.

#### Relevance of Research

Producing research that meets the needs of PMs and funders should help enable the successful adoption of the findings. The notion of conducting policy-relevant research was mentioned by 9 producers and several policy makers. “Relevance” was created if the research (1) aligned with the agendas of decision makers, (2) addressed a current, critical problem, and (3) was interesting to decision makers. In addition, the appropriateness of the evidence produced, especially pertaining to specific research questions, was also an important enabler of its use.

*I think it has to do with the appropriateness of the evidence in question. You can’t compare apples with peppers, or you can’t look at an orange and draw a banana. So, for you to be able to use the evidence … you need to actually have the right evidence for the right question.”* —PM-4

*Sometimes they [RPs] are also on their own, doing their own research that doesn’t fit into what we as a Ministry are looking out for. And so, there is that miscommunication between the RPs and the PMs.* —PM-6

The appropriateness of the evidence produced was an important enabler of its use.

The timeliness of the research also contributed to its relevance. For example, research that addressed the components of the Millennium Development Goals (MDGs) was deemed relevant, as the MDGs are a high-priority policy concern. Being able to provide evidence while a policy is still undergoing review allowed research findings to be included in revisions.

*The best time for influencing policy is when it is under review. Maybe there’s something that was not originally in the policy and you want it to be incorporated in the policy—that’s the best opportunity, when the policy is under review.* —RP-5

To PMs, research was relevant when it fulfilled their needs in a timely way and if the results were made easily available.

*One of the key things is that they [RPs] are doing things that are relevant to the implementers … So then that makes it easy … moving it into the policy.* —PM-1

PMs described the lack of timeliness as a major barrier to using research evidence for policy making. One PM expanded on the issue of policy-making timelines being different from that of academia:

*If [after] 2 weeks the report has not come and in the format that will not pass [be acceptable enough in order] to write some information from it to influence policy, forget about it …So that is how relevant timeliness is when talking about policy foundations.* —PM-2

PMs cited September as a crucial time for PMs in Ghana. By that month, ministries must send their proposed budgets for review before the budgets are read by the Ministry of Finance in November. Thus, institutions should present research findings to PMs by June to give them time to collate the information at the national level. Failing to follow these timelines results in information and recommendations not being incorporated into the budget. Another barrier mentioned by a PM was the failure to involve the communities that would benefit from the health policies under examination.

Respondents mentioned another barrier was the failure to involve the communities that would benefit from the health policies under examination.

When research was commissioned by PMs, it was deemed highly relevant; however, PMs also viewed the costs of commissioning as determining the extent of the research carried out. Ultimately, even where commissioned research findings were useful, the lack of available funding sometimes made research evidence non-implementable.

*Evidence comes at a cost. So, if we have to commission and the cost of evidence to inform our decisions soars up to a point, there’s a limit on how much research you can do before you take action.* —PM-4

*Sometimes it is the cost implications of the interventions—or the decision whether it will be sustainable. … For example, we did some work in the Upper East where we found out that using voice messaging to pregnant women does improve dropout [rates for antenatal attendance] dramatically in a research setting. That was the evidence, but going to scale, we could not afford to send voice messages to all pregnant women of Ghana. Although the evidence was strong … we could not use it as a program because we could not afford it.* —PM-1

PMs’ failure to communicate their specific research needs poses challenges to the eventual adoption of the findings.

*We also have to come out with what our research needs are, what are the things that we want further work to be done on, and then share. So, creating that dialogue platform creates the opportunity to share what the issues are.* —PM-6

This discrepancy is further discussed in the section on the relationship between RPs and PMs.

#### Quantity of Research

PMs considered the availability and access to a breadth of evidence as enabling factors that facilitate policy making. Lack of available evidence required at a given time represented a barrier. One PM discussed how the absence of available research could result in actions being taken based solely on expert opinion, stating:

*The availability of evidence is also fundamental. … So, if the evidence is not available, and we can have expert opinion to take a decision, we’d use expert opinion to take a decision; it’s fast, it’s quick, it may not be too expensive.* —PM-4

PMs also mentioned concerns about duplication of research; in their view, it represents a waste of resources and was usually due to the inaccessibility of existing research conducted previously by academics.

*Sometimes too, some of the things have already been done that you wouldn’t know. You may commission research for somebody to do, but then there might be a lot of evidence that has already been generated elsewhere, sitting somewhere.* —PM-6

### Communicating Findings

The second key factor involved the communication of findings. PMs agreed that research findings must be presented in user-friendly formats, such as concise policy briefs. Respondents joked that they had no interest in seeing documents with *P*-values.

*A policy brief tells you what exactly [the study’s] aim [is] and what they want to achieve and … the language is a bit flexible for us. All the p-values and all of them don’t really … the jargons are limiting. Now, you talk in the language of the PM? … That is what, in my view, makes policy briefs [better]: … the ability to come out with a concise but more informative document.* —PM-2

RPs also mentioned the importance of communicating via simple and understandable means and tailoring their communication to various audiences. Several discussed communication in terms of “engagement.” They reported using different mediums for communication, including holding meetings, participating in policy dia-logues, giving presentations, conducting workshops, writing policy briefs, and engaging with the media.

*Definitely the format—it should be meaningful … So it is the engagement, it is not just the format. It is the way you also engage policy; the way you speak into the issues and the way you also make it relevant to the issue; that will make it acceptable.* —RP-9

Some PMs reportedly do not employ research evidence because they do not find it relevant or do not know how to read and interpret academic reports.

*It’s not everybody in the [parliamentary] committee who has that level of training to appreciate research and to gather data [published research].* —PM-10

RPs discussed the inability to appropriately communicate findings to PMs as an inhibiting factor.

### Strength of RP–PM Relationships

The third key factor, the strength of relationships, reflects perspectives from RPs and PMs on their relationship, as well as RPs’ views on relationships with their fellow producers and other stakeholders.

#### Relationships With PMs

Of 12 RPs, 5 described involving PMs in their research from the beginning stages when developing studies, thus creating opportunities to build relationships. They described organizing sessions to train PMs on various aspects of research to foster engagement and present research findings and their policy implications. One institution reported seconding its staff to policy institutions for limited periods for reciprocal benefits and as a way to facilitate reciprocity.

*To share ideas and in that way, you are learning from them the policy making process and they are also learning from you. … Then together, you come up with a better way.* —RP-5

*I think it is the relationship and the engagement with the program implementers and the PMs. . . .* —RP-11

*It becomes better when the PMs and implementers are involved [in the research] right from the word go, at the beginning…so that you see a smooth transition, you see a desire and interest, to get it going.* —PM-3

Several research producers described involving policy makers in their research from the beginning when developing studies, thus creating opportunities to build relationships.

Some government agencies have their own embedded research institutes that foster research–policy collaborations at various stages. For example, the Ghana Health Service has created an internal research division. This and other government research divisions and institutions are readily available to provide research evidence related to government policy agendas. Another PM also mentioned the government’s research efforts, including that they receive research funds from other donors:

*That’s actually the purpose of setting up the health research center in Navrongo: just to look at the service handicaps. If immunization is going up, why? If there is malnutrition, find out in the area: what is happening? And that’s why [the research centers] were cited in the North, Navrongo, Middle, Kintampo, and then [the South] Dodowa. Now they generate their own funding—it’s not government giving them money because [the centers are] internationally acclaimed.* —PM-1

The involvement of RPs in these efforts extends beyond producing research to working with implementers to carry out evidence-driven programs.

**Opportunities to Engage PMs.** Providing RPs opportunities to engage with PMs was seen as an enabler of using evidence when creating policy.

*The doors of Parliament are open to research. People want information so that decisions they arrive at are well-informed decisions. So don’t hesitate! Always come to Parliament, share what you have, so that research does not gather dust, as research is used to make decisions so that our country can make progress.* —PM-10

Of course, as described above, the process is not quite so simple.

**Making Use of Policy “Champions.”** PMs noted that having a “champion,” an influential person who promoted particular research agendas to influence policy, was also an enabling factor. RPs have leveraged this tactic in the past, leading to the adoption of various national health policies and initiatives.

*You need to present [evidence] in a way that will attract somebody to own it and run with it. You see, somebody very influential to own it and say that “oh, this is something that I’ve been thinking about, this is something that I want to do.”* —PM-1

*The person [RP]… understands the evidence and it appeals to [them] and the [RP] pushes it to somebody to champion it through the policy process. Then the person producing is not in a position to drive it. His … role, I think, ends with the ability to present the evidence in a form that will get that intermediate person who is powerful and influential enough to move it into the policy level.* —PM-1

One PM provided examples of champions who promoted evidence to inform policy:

*The [Community-based Health Planning and Services] was like that. … Dr. Adibo moved it. … And the health insurance was like that to [President] Kufuor*. —PM-1

However, relationships among PMs and RPs faced numerous potential barriers, including the “disconnect” between academics and implementers, or RPs and PMs, in terms of how they relate to, trust, and understand each other. Barriers were also encountered when the evidence produced was not of interest to influential persons who could champion findings into policy.

#### Relationships Among RPs and Other Stakeholders

RPs from 2 institutions mentioned the importance of networking with other organizations that sought to achieve the same goals. Forming a coalition with organizations with similar interests allows them to achieve their unified goals and make a greater impact.

*The network that you are able to build, that is, among civil society organizations … your colleagues. Are you saying the same thing or are you going out alone? That is also very important … that has been a strategy of ours that we use. So you will realize that we also work through a lot of coalitions. So we are members of [a coalition] and we were instrumental in its creation … we work with others through some of these networks.* — RP-7

Other stakeholders can influence the use of research findings in policy development. One RP focused on the importance of engaging with communities, including in the dissemination of findings to ensure research findings truly reflect the needs of the people they sought to serve. Another respondent noted that engaging the media by sharing research findings with them was an effective enabler of promoting the use of evidence for policy making:

*We have found media campaigns to be very effective … when properly mobilized and deployed. And it depends on the partnerships that one builds and the information that you make available to the media. Because we realized that if the media basically highlight or makes it an issue … it also draws attention of PMs. Especially if the framing of the issue in the media could be detrimental to what government expects. So, media advocacy is also very key.* —RP-7

### Structures That Promote Sharing Evidence With PMs

The fourth key factor comprises discussions on institutional and parliamentary structures that support the evidence-based policy process.

#### Institutional Structures

Use of evidence in policy making relies on more than the actions of individual RPs and policy champions. PMs noted that institutional structures can be put in place to enable the research-to-policy process, including: (1) research units embedded in government agencies (such as creating research divisions/units and national health research centers); (2) technical support systems (by engaging people with technical expertise to support the government); and (3) knowledge translation platforms (for example, the National Forum on Health Research, which brings together RPs, PMs, and implementers). These structures provide regular ongoing opportunities for dialogue and collaboration between PMs and RPs. One PM described how they use such structures to bring together various stakeholders into communities of practice:

*The Knowledge Translation Platform [set up by the Ghana Health Service with USAID (United States Agency for International Development) support] is trying to bring together PMs, policy researchers, implementers to be on the same platform. … The National Forum on Health Research is one of such platforms. You know that [with regards to] policy we say, “Come and tell the implementers, the PMs, what they are doing.” And let’s focus on one area, what we call the “community of practice.” We choose community health or … maternal health and look at that, and see “What are the challenges within the country?” And we start to focus on them and then address them and get them into policy.* —PM-3

Use of evidence in policy making relies on more than just the actions of individuals—institutional structures can provide opportunities for dialogue and collaboration.

As stated, institutions embedded as research units within the government “to generate empirical data to support policy” were considered to be “part of the service” (RP-4). As such, their findings are particularly important as the government designs and implements health policies.

These structures, of course, can have their own problems. Barriers related to these structures—such as the “fragmentation of structures,” weak institutions, and “the absence of a national data repository”—were mentioned by one PM, who also recognized that:

*It is very difficult getting data, especially from other ministries, department, and agencies. Even within the Ministry of Health, if it is not a service utilization data, and if it is other data that you need, it’s difficult because we have a lot of agencies and so the fragmentation also makes it a bit difficult.* —PM-5

#### Research Personnel Within Parliamentary Structures

One sign of the recognition of the value of research for policy making in Ghana is that beginning in 2020, parliamentarians have been assigned research assistants with the relevant skills to gather data to support evidence-informed decision making.

*This is the first time Parliament has recruited research assistants to support us in our work, to get the data for us . . . . Parliamentarians have to be well-informed so that they can make intelligent, well-informed decisions.* —PM-10

One barrier discussed was the overwhelming nature of the parliamentarians’ work due to their dual roles in Parliament as lawmakers and serving their constituencies as development workers.

*Our constituency doesn’t judge us by how [we are] formulating policy or taking decisions, [but by] how often we are in the community building bridges, drilling boreholes, and supporting people to get into hospital and issues like that.* —PM-10

Parliamentary research assistants may also end up engaging in activities other than gathering data to inform policy.

*The research assistants in Parliament are to assist the parliamentarian, gather data to support some policy issue that is ongoing, and so that is their duty. And normally they qualify, they come with Masters [degrees] in research, so certainly they are manageable [able to manage] when it comes to their doing data, because that is their job. And so, we try to use them for that purpose. … [But] because of the nature of work of the Ghanaian parliamentarian,* ***the research assistant ends up just doing [running] more errands with the parliamentarian than concentrating on research.*** —PM-10 (emphasis added)

This PM believed that a better organizational structure could support more use of evidence in policy making. In that structure, PMs would have additional support staff, leaving skilled research assistants to focus on their main function: supporting the application of research evidence to lawmaking.

### Political Context in Which Research and Policy Making Occur

As noted previously, expectations of their con-stituents and organizational structures of policy-making bodies affect the capacity of PMs to use research evidence in their policy work. That is, policy is only one part of politics. This final key factor discusses a wide array of other political conditions that influence the use of evidence, including the overall political context, the relationship between health and political priorities, and RPs’ understanding of politics.

Policy is only one part of politics: a wide array of political conditions influence the use of evidence.

#### Political Context

Several PMs noted that political context is a key factor in research utilization. The political environment can enable or inhibit the process of using research when making policy. For example, a study setting must be conducive to enabling evidence generation during certain types of research.

*The political environment must also be quite conducive for research … If [data collection] is in an environment where partisan politics is at play, people will be telling you “Why do you have to come and ask me these questions?”… So there must be some conducive political environment for research activities in the country where you can do your HPSR.* —PM-10

#### Political Manifestos Determine Health Priorities

Another fundamental issue is the focus of the political party in power. The agenda set in the ruling party’s manifestos drives its priorities for health reform. This can be beneficial for specific health causes featured in the manifesto. For example, in 2000, the New Patriotic Party (NPP) manifesto promised to replace the “cash-and-carry” system of health payment with a national health insurance scheme. Once the party was elected, President Kufuor championed this policy, which was based on research conducted at a national health center.^14^

However, some PMs who worked at implementing agencies noted that most manifesto promises were not based on research, as politicians did not consult those with technical knowledge from the outset.

*But … the promises that they make are normally not based on research … [although] every political promise should be based on research.* —PM-8

*For politics, it’s about winning power, and there we also have a government that may probably have an agenda, but we also have a manifesto. The political manifesto is different from the national technical [plan]. . . . When manifestos are being formulated, nobody gets in touch with the technical strain. So, when you come to power then you have a manifesto which people will hold you accountable to for the next election.* —PM-3

If research supports the government’s political priorities, the RPs and their institutions can benefit. Alignment with the government can result in government financial support for research. One PM noted, however, that lack of alignment can also harm RPs:

*Now you are getting into the political environment, politics of the left and the right. If the politics [of the research agenda] does not favor the right, and the right is in government, then you won’t get it through.* —PM-3

Another PM articulated this issue in the following exchange with an interviewer:

PM-2: *You see, you want implementation, and who provides the resources for implementation?*

Interviewer*: The government in power.*

PM-2: *Yes, and the decision to allocate resources … they decide that “this amount is going to Ghana Health Service, this is going to other agencies.” … That is why when we are doing or formulating review policy, we always want the Ministry of Health to come in. The reason is, when it comes to providing the wherewithal, the funds to implement, they make the political decision.*

#### Understanding Politics in Policy Making

PMs perceived that RPs do not fully understand politics and tend to behave in ways that “[distance] them from reality” (PM-1), leading PMs to distrust RPs’ viewpoints.

*And also, [RPs] not understanding the politics of policy. You see, because policy goes beyond just evidence, so sometimes they are very naïve in their thinking. … There are a lot of counterfactuals that need to be looked at, and if they understand that one, then I think it will inform the way they do their communication. Yeah, that they understand the power games at play and the stakeholders … involved in all the processes. I think sometimes they are limited in their understanding of that.* —PM-1

Notably, RPs did not explicitly discuss the issue of politics in the ways that PMs did. One RP suggested the need for political connections because, “you can only influence policy when you are part of the inner cycle of the policy makers” (RP-2). Organizations involved in advocacy expressed an understanding that they need to forge relationships with PMs and implementing institutions to conduct relevant research and tailor appropriate messaging to influence policy.

## DISCUSSION

We used qualitative information from interviews with HPSR producers and PMs in Ghana to identify enablers and inhibitors of evidence-based policy making. For the most part, the enablers and in-hibitors identified by each group were similar. The 2 groups also provided comparable accounts of how policy could be informed by evidence. Themes in line with components of the PARIHS framework include the quality of research evidence, nature of the context, and relationships with facilitators.

A key thread running through both groups’ understanding of how research evidence gets incorporated into policy making was the importance of involving PMs in the research process—suggesting an incremental interactive or enlightenment model of communication between the groups.^26^^,^^27^ This emphasis highlights the importance of involving PMs not only in the process of building and strengthening relationships but also in determining what research evidence should be produced and how to appropriately communicate findings.

Relationships between the 2 groups were repeatedly cited as one of the main drivers of research uptake. Building relationships between PMs and RPs allows for collaborations, mutual understanding, and the evolution of trust, which in turn allow for greater uptake of evidence. Similarly, the disconnect between research and policy (referred to as a “gulf” between RPs and decision makers by Orton et al.^28^) was partly attributed to a mutual lack of trust, among other factors. Particularly, in the absence of structures that promote RP-PM relationships and engagement, PMs may question whether RPs have a hidden agenda when presenting their findings. Alternatively, involving PMs in a research effort makes the process transparent and builds PMs’ trust in research findings.^29^ This interactive/enlightenment model in the research-to-policy process was carried out in the case of Ghana’s Community-Based Health Planning and Services initiative, among other similar successful national health programs.^12^ It must be noted that a symbiotic relationship between the RP and PM would not compromise the principle of scientific independence as long as RPs are guided by the ethical principles of objectivity, integrity, and truthfulness.

Relationships between RPs and PMs were repeatedly cited as one of the main drivers of research uptake.

Relevance of the research conducted to PMs’ concerns is another factor tied in with PM involvement. Involving PMs from the start is one way for RPs to frame timely research questions that apply to policies under consideration. PMs stated that they were open to providing opportunities for RPs to engage with them and to promote uptake. This view was expressed, for example, by a member of Parliament, who indicated appreciation of health research in informing the law-making process. Government-commissioned research represents another (albeit costly for the government) opportunity for RPs to partner with selected institutions. Therefore, RPs typically sought international funding opportunities. Hennink and Stephenson^26^ describe this as a “tactical model” used to inform policy with research commissioned on topical issues.

Interviewees also cited the choice and execution of means of communication as important factors in PM involvement. The key communication challenge is that research findings tend to be disseminated in overly technical language; PMs prefer simplified summaries of research findings. One benefit of long-term collaboration between RPs and PMs is that both parties learn to “speak each other’s language.” Further, such collaboration enables PMs to track emerging research findings, rather than having to wait for end-of-project dissemination activities. PMs interviewed rarely mentioned academic journal articles as a source of evidence for decision making. However, these articles are the primary way that RPs disseminate their findings and play a key role for those working in academic institutions, where career progression is tied to peer-reviewed publication. Therefore, RPs and advocacy organizations should invest time and effort in developing modes of communicating that are more acceptable to PMs, such as hosting meetings, participating in policy dialogues, giving presentations, conducting workshops, preparing policy briefs, and engaging with the media. According to Elliott and Popay,^27^ these activities, conducted by RPs, allow for a “common language” to develop between the 2 groups.

The key communication challenge is that research findings tend to be disseminated in overly technical language; PMs prefer simplified summaries of research findings.

While increasing PM involvement in research should contribute to the use of evidence in policy development, it also comes with risks. One risk is the potential for politicization of research to support purely political agendas. This possibility raises concerns about the involvement of politicians in the research process, as they may seek to exert influence on how the research is conducted or interpreted to produce politically appropriate findings. In Ghana’s democracy, where political parties can be voted out of power after 4 years, politically driven policies could lead to lack of commitment to policies implemented by previous governments, regardless of the evidence that supports them. A tendency to halt predecessors’ evidence-based interventions is especially worrying considering the financial cost, time invested, and other resources RPs and implementers use to contribute to health policies.

### Implications of Findings for Future Implementation

Our findings have potential implications for implementation research, especially in LMICs. First, RPs should, to the extent possible, engage PMs in the design and implementation of their research to ensure that the research is relevant to and feasible for program implementation. This engagement should also enlighten RPs on contextual factors, including available institutional structures, systems, and procedures to facilitate policy making and the politics involved in the process. Second, to ensure that research finds relevance among PMs, RPs should build into their dissemination agenda the production of policy briefs and tailored project reports that PMs can readily comprehend. These initial actions are especially well-suited to addressing challenges that plague LMICs, helping to ensure appropriate outcomes in such settings.

### Limitations

The main study limitation was the busy schedules of the respondents and bureaucratic requirements of some organizations, particularly after the start of the COVID-19 pandemic, forcing interviews to be postponed or canceled. To minimize these challenges, we provided participants with soft copies of the interview guide and conducted phone interviews where possible.

Another major study limitation stems from our inability to interview all RPs and PMs identified as relevant for the study. The PMs we interviewed were bureaucrats from health ministries, agencies, and institutions. Thus, we cannot generalize findings from this qualitative study to PMs from other sectors, positionings, or contexts with divergent processes or dissimilar health policy agendas from those in Ghana. Therefore, further research is required to understand these important issues among a range of policy decision-making actors. Further, LMICs continue to lag behind other countries in the uptake of health evidence. Weak health systems ensue, and innovations and funds to improve the health system are nonexistent. Despite these limitations, our findings are useful for understanding enablers and inhibitors of the health policy process in the Ghanaian context—offering contributions to its successes and challenges as uptake of the practice intensifies.

## CONCLUSION

We highlight several factors enabling or inhibiting evidence-based, health-policy decision making in Ghana and potentially in other LMICs with comparable health policy contexts. This study identified 5 specific factors that can enable or inhibit the process of translating health research into policy in Ghana: (1) the quality, relevance, and quantity of research evidence; (2) how findings are communicated to PMs; (3) the strength of relationships between PMs and RPs; (4) available institutional and personnel structures that promote the use of research in policy making; and (5) the wider political context and conditions in which research and policy making occur. Literature on factors in the research-to-policy process indicates some themes similar to ours—specifically regarding the research produced, how it is communicated, and RP–PM relationships. However, previous studies offer less discussion on the structures required for a smoother research-to-policy process and on the role of advocacy in the political arena to promote the use of research for policy formulation.

Our findings point to a need for RPs to consider PMs’ evidence needs throughout the research process. RPs also need to ensure they are producing timely and relevant research outputs and to accommodate PMs’ preferred communication approaches. Thus, RPs need more support and capacity development to translate their research findings into language that is more accessible to PMs, as well as present their evidence in formats that are readily usable by non-experts. RPs and PMs also ought to collaborate more in setting research agendas and even in defining research questions. RP-PM collaborations can bridge research with real world decision making, thereby promoting opportunities for translation of evidence into policy. In addition, RP-PM collaborations can help research teams craft context-appropriate policy recommendations that are cost-effective, affordable, and able to be implemented under current conditions.

## Supplementary Material

GHSP-D-21-00693-supplement.pdf

## Notes

**Peer Reviewed**
