# Supplementary material for: “You Can’t Look at an Orange and Draw a Banana”: Using Research Evidence to Develop Relevant Health Policy in Ghana
Source: Glob Health Sci Pract. 2022 Sep 15;10(Suppl 1):e2100693. doi: 10.9745/GHSP-D-21-00693 (PMC9476488; doi:10.9745/GHSP-D-21-00693)
Supplement: GHSP-D-21-00693-supplement.pdf [file GHSP-D-21-00693-supplement.pdf]

Supplement to: Bawah AA, Biney AAE, Kyei P. "You can't look at an orange and draw a banana": Using research evidence to develop relevant health policy in Ghana. *Glob Health Sci Pract*. 2022;10(Suppl 1):e2100693.
https://doi.org/10.9745/GHSP-D-21-00693

HPSR Capacity Assessment: HPSR Producers Interview Guideline

---

1. Reflecting on the times when your research institution has been able to influence policy, what do you think were the principal enablers of this?
   - Probes
     - Address relevant policy question
     - Timing and interest of decision maker
     - Quality of the research
     - The format in which it was presented
     - The venues in which it was presented, including policy dialogues
     - Relationship between you or your institution and the decision-maker, formal and informal
     - The credibility of your institution

2. What is it about your institutional characteristic/profile that allows you to generate high quality policy relevant research and influence policy?
   - Probes
     - Positioning within circle of policy influence
     - Relationship with MOH, both formal and informal
     - The way you identify research priorities
     - Human Resource policies including researcher recruitment and retention policies. This includes providing researchers a clear career track
     - Funding arrangements, both source as well as level of financial autonomy, such as ability to determine researcher salaries, and draw in project funds

3. How has your organisation developed and sustained capacity for HPSR? What are your strengths and weaknesses? And what factors can speed up your HPSR capacity?
   - Probes
     - Human resource and skill-mix & challenges
     - Adequate funding and its sources
     - Supporting facilities and environments

4. Please give notable examples of your contribution to the national/international health policies

Supplement to: Bawah AA, Biney AAE, Kyei P. "You can't look at an orange and draw a banana": Using research evidence to develop relevant health policy in Ghana. *Glob Health Sci Pract*. 2022;10(Suppl 1):e2100693. https://doi.org/10.9745/GHSP-D-21-00693

HPSR Capacity Assessment: Policymakers Interview Guideline

----

1. Evidence-based policy environment in your country

1.1 HPSR demand & utilization

- How often do you use research evidence to guide policy decisions? What situations would you use or not use evidence in policymaking? Why?
- What are the culture and trend of evidence-based informed policy in health in your country? How have they changed over time? Why?
- What is the most suitable format of research products that can promote policy uptake? Please explain the reasons for your answer.
- In order to stimulate the culture of using HPSR evidence to guide policy implementation; program performance or impact assessment contributed by HPSR is critical. In your country, is program evaluation a) a mandatory by law or policy with strong adherence, b) mandatory by law or policy but not strictly adhere to, c) non-mandatory but commonly practice, d) not at all practice

1.2 HPSR supply

- Is the number of HPSR researchers/institutes sufficient to satisfy HPSR demand by policy makers? If so, why?
- What are a) enabling factors, b) barriers in getting and using evidence to policy?
- Probes
  - Relationship and linkage between policy makers and HPSR researchers/institutes
  - The source of research evidence
  - Trustworthiness of research evidence
  - Relevance to policy questions and country's problems
  - Timeliness of research to answer policy requests
  - Feasibility and actionability of policy recommendations
- If there are any research institutes that you trust or rely on, why, what competency that they have? (e.g. their formal/informal linkage to policy makers, global/domestic creditability)
